# Importance of Spatial Arrangement of Cardiomyocyte Network for Precise and Stable On-Chip Predictive Cardiotoxicity Measurement

**DOI:** 10.3390/mi14040854

**Published:** 2023-04-14

**Authors:** Kazufumi Sakamoto, Suguru Matsumoto, Nanami Abe, Mitsuru Sentoku, Kenji Yasuda

**Affiliations:** 1 Department of Pure and Applied Physics, Graduate School of Advanced Science and Engineering, Waseda University, Tokyo 169-8555, Japan; 2 Department of Physics, School of Advanced Science and Engineering, Waseda University, 3-4-1 Okubo, Shinjuku, Tokyo 169-8555, Japan

**Keywords:** cardiomyocyte, predictive toxicity, synchronization, geometry dependence, community effect

## Abstract

One of the advantages of human stem cell-derived cell-based preclinical screening is the reduction of the false negative/positive misjudgment of lead compounds for predicting their effectiveness and risks during the early stage of development. However, as the community effect of cells was neglected in the conventional single cell-based in vitro screening, the potential difference in results caused by the cell number and their spatial arrangement differences has not yet been sufficiently evaluated. Here, we have investigated the effect of the community size and spatial arrangement difference for cardiomyocyte network response against the proarrhythmic compounds from the viewpoint of in vitro cardiotoxicity. Using three different typical types of cell networks of cardiomyocytes, small cluster, large square sheet, and large closed-loop sheet were formed in shaped agarose microchambers fabricated on a multielectrode array chip simultaneously, and their responses were compared against the proarrhythmic compound, E-4031. The interspike intervals (ISIs) in large square sheets and closed-loop sheets were durable and maintained stable against E-4031 even at a high dose of 100 nM. In contrast, those in the small cluster, which fluctuated even without E-4031, acquired stable beating reflecting the antiarrhythmic efficacy of E-4031 from a 10 nM medium dose administration. The repolarization index, field potential duration (FPD), was prolonged in closed-loop sheets with 10 nM E-4031, even though small clusters and large sheets remained normal at this concentration. Moreover, FPDs of large sheets were the most durable against E-4031 among the three geometries of cardiomyocyte networks. The results showed the apparent spatial arrangement dependence on the stability of their interspike intervals, and FPD prolongation, indicating the importance of the geometry control of cell networks for representing the appropriate response of cardiomyocytes against the adequate amount of compounds for in vitro ion channel measurement.

## 1. Introduction

Evaluating the risk of lethal arrhythmia is one of the most critical safety issues in predictive cardiotoxicity for drug discovery technology developments. In drug discovery and toxicology, the International Council for Harmonisation of Technical Requirements for Pharmaceuticals for Human Use (ICH) regulations S7B and E14 request a pair of in vitro ion channel-measurement-based studies and in vivo animal QT prolongation tests to exclude torsadogeneic compounds [1,2,3,4]. The ICH S7B and E14 guidelines have been successful in preventing the introduction of potentially torsadogenic drugs to the market by focusing on hERG blocking and QT prolongation as essential determinants of proarrhythmia risk. However, they still contain overestimation or underestimation for drug development. Hence, the Comprehensive in Vitro Proarrhythmia Assay (CiPA) initiative was established to develop a new paradigm for assessing proarrhythmic risk, building on the emergence of new technologies and an expanded understanding of torsadogenic mechanisms beyond hERG block. They evaluated drug effects on three strategies; (1) human ventricular ionic channel currents in heterologous expression systems, (2) in silico integration of cellular electrophysiologic effects based on ionic current effects, the ion channel effects, and (3) fully integrated biological systems (stem-cell-derived cardiac myocytes and the human ECG) [5].

To improve the clinical relevance in the in vitro screening, the establishment of human stem cell-derived cardiomyocytes (hsCM), which express whole physiologically functioning ion channels of humans, has enabled us to use a more appropriate cell source for assessing proarrhythmic [6,7]. Moreover, human-induced pluripotent stem cell-derived cardiomyocytes (hiPS-CMs) have added significant advantages to generating panels of cell lines that more closely reflect the genetic diversity of a population, such as familial cardiomyopathy, lethal familial arrhythmias, and congenital heart diseases [8,9,10]. However, to date, there has been no report in the conventional in vitro cardiotoxicity assay on the difference of the cell responses depending on their spatial arrangements, which is essential to predict the risk of the compounds quantitatively.

In this paper, we investigate the effect of the proarrhythmic compound, E-4031 on the typical three types of cardiomyocyte networks, small cluster, two-dimensional sheet, and closed loop, formed in those shaped agarose microchambers fabricated on a multielectrode array chip simultaneously. We have described a significant difference in the stability of beating intervals, field potential duration (FPD) prolongation, and temporal fluctuation of FPD in the multi-electrode array (MEA) assay on the three geometry types of different cardiomyocyte networks against the administration of E-4031. We found that each community had the characteristic rate and stability of beating rhythm, consisting of the cardiomyocytes from the same source. In addition, they showed a different response to the administration of E-4031, and the large two-dimensional sheet had a higher tolerance to the drug than the other. These reactions were reversible by eliminating the drug. The results indicate that considering the appropriate spatial pattern on the cell network would be necessary for representing the proper responses similar to clinical results against the number of compounds.

This is the first report evaluating the cell number and their spatial geometry dependence in FPD prolongation and temporal fluctuation of FPD in the MEA assay using hsCMs with representative proarrhythmic compound, E-4031. The importance of precise geometry and number control of the cardiomyocyte network was proposed.

## 2. Principle

In our previous report, we presented the relationship between a single cardiomyocyte’s cellular and extracellular potentials from the results of simultaneous measurements [11]. The extracellular field potential (FP) is the electrical potential of cardiomyocytes caused by the inward and outward ion currents on the cell membrane. In electrophysiological conventions, a negative current value or downward deflection of a current trace on cell membranes is typically referred to as an inward current, reflecting either the movement of positive or negative ions out of the cell. Conversely, a positive current value or upward deflection of the current trace (outward current) also reflects either the movement of positive ions out of the cell or negative ions into the cell through the cell membrane. Action potential (AP) occurs when the membrane potential of a cell location rapidly rises and falls caused by the rapid change of the net charge in the cell. As the origin of FP for individual cells is the electrical potential difference across the plasma membrane of excitable cells caused by the cells’ inward/outward ion currents, the time course of FP, $V_{f}$, is proportional to the transmembrane ion current of cells, $I_{ion}$, which is the AP time differential, $\phi_{a}$, of cells. The relationship of $V_{f}$ to $\phi_{a}$ is theoretically described as,

(1) $$V_{f}=I_{ion}R=R\frac{d\phi_{a}}{dt}$$

where *R* is the resistance of the environment and measurement system, and time course $V_{f}$ amplitude is the value of the FP microelectrode measurement.

APs can distinguish phenotypes and electrophysiological changes in cardiomyocytes in conventional patch-clamp studies [12]. The time differential of those APs can estimate FP waveforms of phenotypes applying Equation (1). The experimental results of FPs can also be used to distinguish phenotypes and electrophysiological changes similar to their time differentials of APs. In practice, FPs can also be used for long-term screening of the electrophysiological properties of cells on microelectrodes, which can overcome the limitations of experiment time and simultaneous measurement number of cells.

Hence, we used differences in FPs due to differences in the spatial arrangement of the cardiomyocyte network as an indicator for this study. The FPs results may likewise suggest differences in APs due to differences in the spatial arrangement of the cardiomyocyte network.

## 3. Materials and Methods

### 3.1. Agarose Microchamber Formation on Multielectrode Chips

The agarose microchambers on the multi-electrode array (MEA) chip (MED-P545A, AlphaMED scientific, Osaka, Japan) were prepared as follows. First, the MEA chip was hydrophilized with a plasma ion source device (PIB-20, VACUUM DEVICE, Ibaraki, Japan). Then, the MEA chip was coated with collagen (Cellmatrix Type I-C, KP-4100, Nitta Gelatin, Osaka, Japan) diluted ten times with 1 mM HCl. After drying for 30 min at room temperature, the MEA chip was covered with a 3% agarose (Agarose Low melting point analytical grade, v2111, Promega, Madison, WI, USA) with the spin coater (1H-D7, MIKASA, Tokyo, Japan). The covered thin agarose layer was melted by the spot heating of 1480 nm focused infrared laser (RLM-1-1480, IPG Laser GmBH, Oxford, MA, USA) to form the three types of two-dimensional format: small sheet, square large sheet and square closed loop structures were fabricated.

### 3.2. Human Embryonic Stem Cell-Derived Cardiomyocyte Cultivation

Human embryonic stem (hES) cell-derived cardiomyocytes (hES-CMCTM002, hES cell line SA002) were purchased from Cellectis (Gothenburg, Sweden). The hES cardiomyocytes were cultured on collagen-coated 60 mm tissue culture dishes (1.2–2.0 × 10^6^ cells/dish) in culture medium (Dulbecco’s modified eagle medium (DMEM, 11995-065, Thermo Fisher Scientific Inc., Waltham, MA, USA) supplemented 20% heat-inactivated Fetal Bovine Serum (FBS, Thermo Fisher Scientific Inc., Waltham, MA, USA), 2 mM Gultamax (Thermo Fisher Scientific Inc., Waltham, MA, USA), 1% non-essential amino acids solution (MEM NEAA (100X), Thermo Fisher Scientific Inc., Waltham, MA, USA), 0.1 mM 2-mercaptoethanol (Thermo Fisher Scientific Inc., Waltham, MA, USA), 100 U /mL penicillin and 0.1 mg/mL streptomycin) at 37 °C in 5% CO_2_.

After three days in culture, the dishes were rinsed with phosphate-buffered saline (PBS, WAKO, Osaka, Japan). For cell collection, 2 mL of 0.25% trypsin-1 mM EDTA (WAKO, Osaka, Japan) were added to the dishes and incubated for 4 min at 37 °C in 5% CO_2_. Then, the solutions in the dishes were collected and centrifuged at 180× *g* for 5 min. After cell counting, the culture medium was added to 5 × 10^5^ cells/mL. Next, a hundred microlitter of the cell suspensions were dropped two times into each microstructure on the MEA chips filled with 1 mL of culture medium, and the MEA chips were incubated at 37 °C for five hours. Then, the culture medium was exchanged mildly and incubated for two days. The cardiomyocyte networks were cultured for seven days in microchambers on MEA chips in culture medium at 37 °C in 5% CO_2_ while exchanging the medium every day.

### 3.3. Test Compound

We chose the human ether-a-go-go-related gene (hERG) ion channel inhibitor, E-4031 (E-4031 n-Hydreate, WAKO, Osaka, Japan), as the test compound and applied it for experiments [13,14,15]. E-4031 was dissolved in PBS and stocked at 50 mM concentration. For test concentrations (1 nM, 10 nM, 100 nM, 1 μM), the stock concentration was diluted in the culture medium.

### 3.4. Field Potential Recordings

Extracellular field potential (FP) recordings of the hES cardiomyocyte networks were performed using the self-made on-chip MEA system at a sampling rate of 50 kHz with a low path filter of 2 kHz and high path filter of 10 Hz, and amplified by 5000 using the analog amplifier before A/D conversion. All measurements were performed at 37 °C.

First, the MEA chip was placed in the holder of the on-chip MEA measurement system, and the culture medium in the MEA chip was half exchanged. Then, the MEA chip was equilibrated for 5 min, and the FP waveforms were recorded for 5 min in the culture medium. After recording, the culture medium was half exchanged to become 0.1% PBS solution in the culture medium as the control state, and the FP waveforms were recorded in the same ways. Next, the test compound, E-4031, was applied to half exchange the medium to 1 nM, 10 nM, 100 nM and 1 μM concentration sequentially, and the FP waveforms were recorded for 5 min at each concentration. Finally, all medium was exchanged for the fresh culture medium after washing. After 120 min from a wash-out, the FPs were recorded for 5 min as the recovery state.

### 3.5. Data Analysis

The signals of FP waveforms were analyzed with MATLAB (Version 2020b. The MathWorks, Inc., Natick, MA, USA). The data from the last 50 waveforms of the 5 min FP recording were used for each concentration’s inter-spike interval (ISI) and FP duration (FPD) measurement. The ISI was the first peak interval time to the following waveform’s first peak. As described in **Principle** [11], the FPD was defined as the duration time between the first peak of the inward current of depolarization, mainly caused by sodium ion channels, and the following last peak of outward current of repolarization, mainly driven by potassium ion channels, of a waveform. Hence, we measured these two peak times of FPDs to be the same as the measurements of APs in the conventional patch-clamp measurements.

The coefficient of variability (CV) of ISI was calculated as,

(2) $$CV_{ISI}\left(\%\right)=\frac{\mu }{\sigma }\times 100$$

where, the ratio of the standard deviation σ(s) to the mean value μ(s) of IBI. To evaluate the stability and reproducibility of electrophysiological cell response in their spontaneous firing intervals quantitatively, the short-term variability (STV) of FPD was also calculated as,

(3) $$STV_{FPD}\left(ms\right)=\frac{1}{N\sqrt{2}}\sum_{k=1}^{N}|FPD_{k+1}-FPD_{k}|$$

where $FPD_{k}$ represents the FPD of beat *k*-th, and N+1=50.

### 3.6. Statistical Analysis

All values are presented as mean ± standard deviation (S.D.). Dunnett’s multiple comparison tests performed drug effects at each concentration to compare with the control state. Tukey’s multiple comparison tests conducted drug effects between cell network patterns to compare groups with each other. *p* < 0.05 was considered significant. R (version 4.2.1 (2022), R Core Team, GNU) analyzed statistics.

## 4. Results and Discussion

### 4.1. Fabrication of Three Formats of Cardiomyocyte Cell Network Patterns

**Figure 1:**
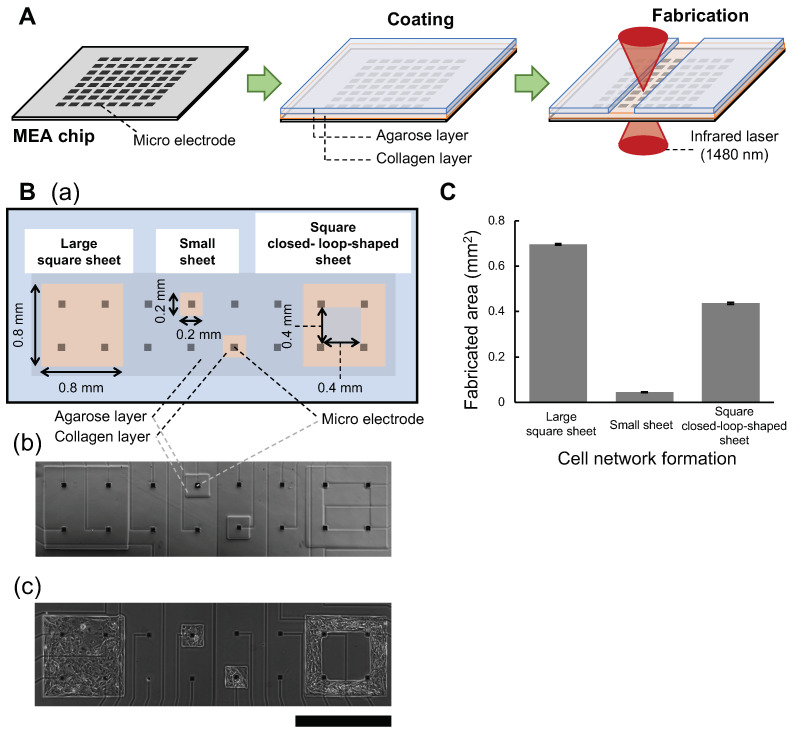
**Fabrication of three types of cardiomyocyte networks on a multielectrode chip.** (**A**): Schematic drawing of fabrication procedure of agarose microchambers on a multi-electrode array (MEA) chip with photo-thermal agarose microfabrication technology. An MEA chip was coated with collagen and agarose layers. Then a portion of the agarose layer was melted by the spot heating of the focused infrared laser. (**B**): three shapes of three types of agarose microchamber structures for cardiomyocyte networks formed on an MEA chip. (**a**): schematic drawing of the agarose microchamber designs for cardiomyocyte network on an MEA chip. (**b**): a micrograph of the fabricated three shapes agarose microchambers on the one-quarter area of an MEA chip; large square sheet, small sheet, and square closed-loop-shaped sheet. (**c**): a micrograph of agarose microchambers on the same one-quarter area of an MEA chip two days after human embryonic stem (hES) cell-derived cardiomyocyte cultivation. Bar, 1 mm. (**C**): The size distribution of three types of agarose microchambers on an MEA chip. Bars and error bars represent the mean and S.D. of agarose microchamber areas, respectively. (n = 3).

To evaluate the dependence of spatial arrangement differences of cardiomyocyte network against the compounds, we have designed three types of networks on a chip: the large square sheet, which mimics the homogeneous two-dimensional (2D) cardiomyocyte network as a sheet; the small sheet, which mimics the isolated or lower connected cardiomyocyte network or a small cluster; and the square closed-loop-shaped sheet, which mimics the micro reentry pathways and the shape of the contour of the square closed loop was the same as the shape of the large square sheet. We fabricated these three types of agarose microchambers on an MEA chip with agarose microfabrication technology (Figure 1A) and cultivated cardiomyocytes in each microchamber simultaneously to allow for simultaneous comparative measurements of these three type cardiomyocyte networks under the same cultivation and medium conditions.

Figure 1B(a) shows a schematic drawing of the formats of three types of agarose microchambers for cardiomyocyte networks on an MEA chip; the microchamber for large square sheet (0.8 × 0.8 mm square shape design), the microchamber for small sheet (0.2 × 0.2 mm square shape design), and the microchamber for square closed-loop-shaped sheet (0.8 × 0.8 mm) outer square shape with 0.4 × 0.4 mm inner square shape.

We sowed cardiomyocytes into the three types of agarose microchambers on an MEA chip. Figure 1B(b,c) are the micrographs of the same one-quarter area of an MEA chip before and two days after the cultivation of cells, respectively. As shown in the micrographs, the cells formed complete 2D networks in the microchambers that conformed to the shapes of these agarose containers.

The resolution and preciseness of the photo-thermal agarose microfabrication method for constructing agarose microchambers on MEA chips were evaluated. The fabricated sizes of three large square sheets, three small sheets, and three square closed-loop-shaped sheets on an MEA chip were as follows: large square sheets, 0.696 ± 0.004 mm^2^ (n = 3); small sheet, 0.045 ± 0.001 mm^2^ (n = 3); and square closed-loop-shaped sheets: 0.437 ± 0.005 mm^2^ (n = 3), respectively (Figure 1C). As shown in those results, the photo-thermal agarose microfabrication method is precise enough to fabricate the exact shapes of agarose microchambers on an MEA chip with single-cell size accuracy.

**Figure 2:**
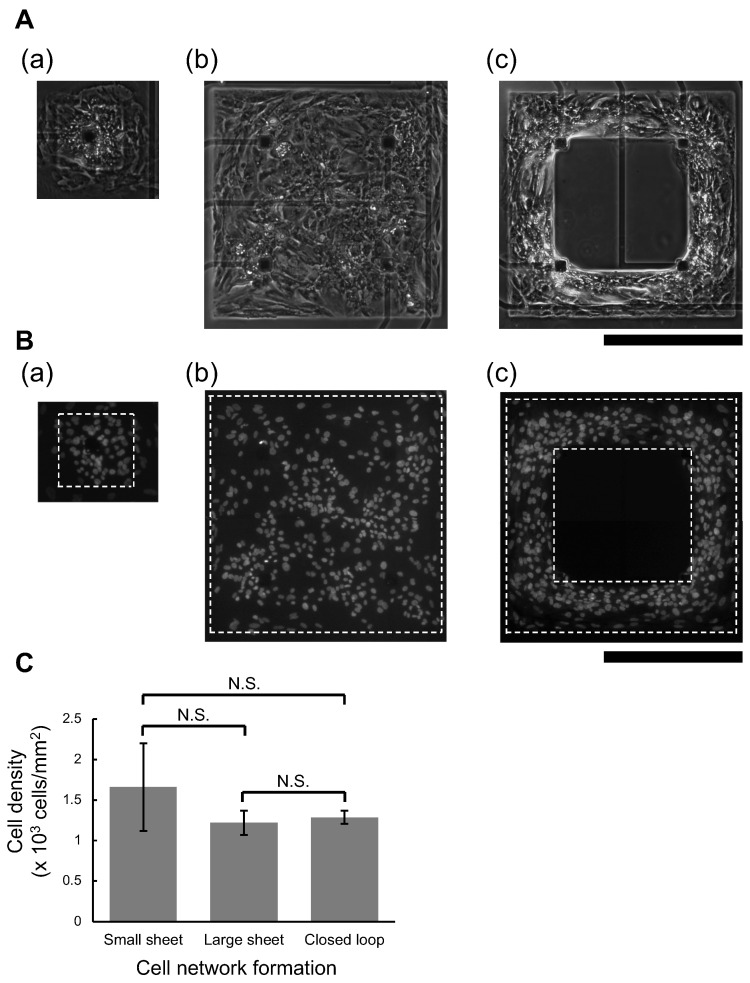
**Cultured cardiomyocyte networks in three patterns of agarose microstructures.** (**A**): Representative micrographs of three different spatial cell arraignment types of hES cardiomyocyte networks cultured on an MEA chip for seven days. (**a**): cardiomyocyte network in small sheet shape agarose microchamber. (**b**): cardiomyocyte network in large square sheet shape agarose microchamber. (**c**): cardiomyocyte network in square closed-loop-shaped sheet shape agarose microchamber. Bars, 0.5 mm. (**B**): representative fluorescent micrographs of hES cardiomyocyte networks. **B**(**a**–**c**) correspond to stained of the nuclei of **A**(**a**–**c**), respectively. The nucleus of each cell was stained by nuclear staining fluorescent dye, DAPI, after the measurement of extracellular field potentials (FPs). Bars, 0.5 mm. (**C**): cell densities of hES cardiomyocyte networks in three patterns of agarose microchambers (n = 3 each). Bars and error bars represent mean values and S.D. in three patterns, respectively. No significant differences (N.S.) were observed between groups.

The spatial distribution of cardiomyocytes in those three types of agarose microchambers was also examined. Figure 2A shows the representative phase-contrast images of cardiomyocytes in the three types of agarose microchambers seven days after the cultivation started; (a) small sheet, (b) large square sheet, and (c) square closed-loop-shape, respectively. Figure 2B also shows fluorescent images of DAPI-stained nuclei of the same samples in Figure 2A. Cell density was obtained by counting the spatial distribution of DAPI-stained nuclei in each agarose microchamber. The cell densities of the small sheet, the large square sheet, and the squared closed-loop-shape sheet were 1.7 ± 0.5 × 10^3^ cells/mm^2^ (n = 3), 1.2 ± 0.2 × 10^3^ cells/mm^2^ (n = 3), and 1.3 ± 0.1 × 10^3^ cells/mm^2^ (n = 3), respectively. As shown in Figure 2C, the results of the statistical tests did not reveal any significant differences in cell density between the groups.

### 4.2. Measurement of hERG Ion Channel Blocker E-4031 Response in Three Formats of Cardiomyocyte Networks

As described in **Introduction**, ICH regulation S7B requests a pair of in vitro ion channel measurement and in vivo animal QT prolongation tests to exclude torsadogeneic compounds [1,2,3]. Significantly, the most famous target ion channel in the former in vitro ion channel measurement is the human-specific hERG ion channel. However, we observed the difference in ion channel responses between single isolated cells and cell clusters in our previous in vitro patch-clamp measurement [12]. Hence, in this report, we focused on the hERG ion channel behavior in three different geometry of cardiomyocyte networks using an hERG-specific inhibitor E-4031. As the conventional in vitro ion channel measurement neglects the effect of cell networking, it is crucial to confirm whether the network formation of cardiomyocytes and their geometry difference affect hERG ion channel response even the most common in vitro hERG inhibitor compound. Therefore, if the dependence of cardiomyocyte network geometry is observed in E-4031, we cannot neglect the cell network size and arrangements even in in vitro ion channel measurements, and furthermore, they need to be aligned carefully for more precise and reproducible in vitro ion channel measurements.

**Figure 3:**
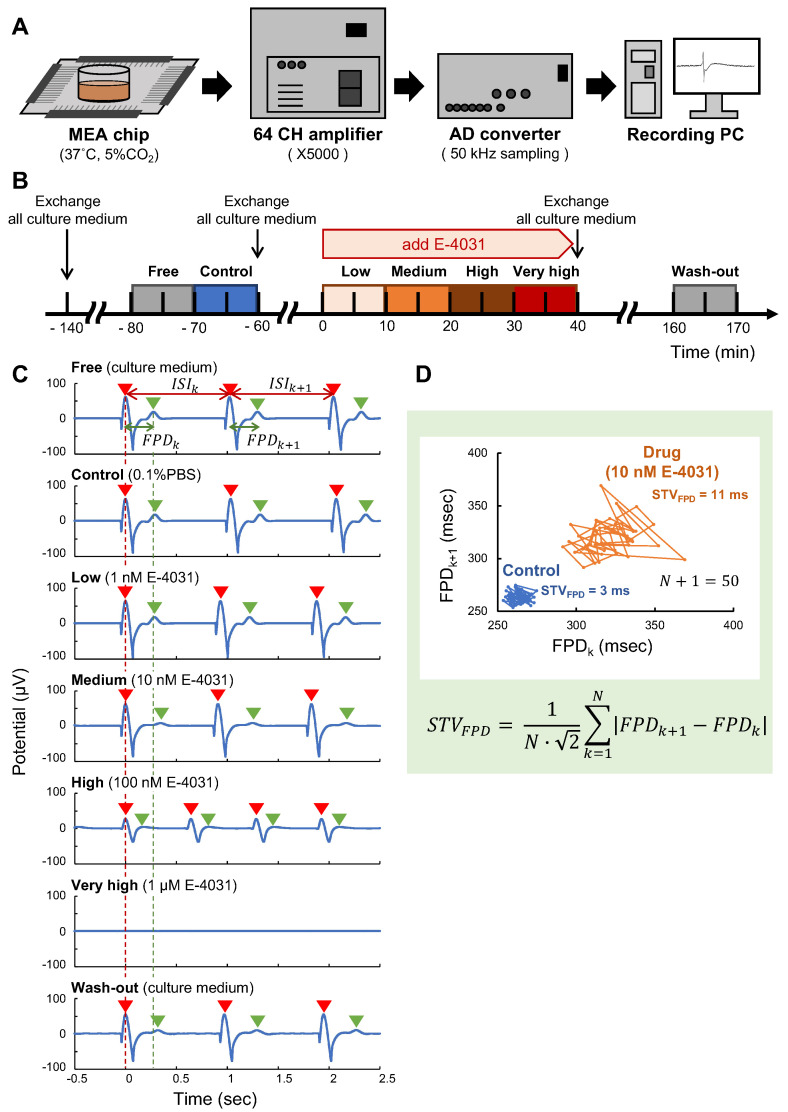
**Experimental designs of extracellular field potential measurement of cardiomyocyte networks: Evaluation of hERG ion channel blocking with E-4031.** (**A**): schematic drawing of the MEA measurement system. The extracellular field potentials (FPs) of cardiomyocytes on the microelectrodes were amplified and digitally recorded for FP waveform analysis. (**B**): time-course of drug administration procedure. The FPs of cardiomyocytes on a microelectrode were measured at seven different solution states in MEA chips: **Free** (only culture medium), **Control** (0.1% PBS dose), **Low** (1 nM E-4031 dose), **Medium** (10 nM E-4031 dose), **High** (100 nM E-4031 dose), **Very high** (1 μM E-4031 dose) and **Wash-out** (only culture medium). (**C**): representative FP waveforms of large square sheet cardiomyocyte network at every seven states. The red and green arrowheads indicate each FP waveform’s first and last FP peaks, respectively. The FPD prolongation, an increase of the time distance of those first and last FP peaks, was observed at the **Medium** state. The fibrillation-like weak and faster ISI waveform was observed at **High** state. An arrest of FP waveforms was recorded at **Very high** condition. (**D**): The evaluation of FPD fluctuation caused by hERG ion channel blocking effect with E-4031 of cardiomyocyte networks. The Poincaré plots of FPDs in Control (blue, STV = 3 ms) and Medium (Orange, STV = 11 ms) states were described in the graph. These visualized fluctuations of FPD were evaluated and quantified by calculating the short-term variability (STV) of FPD.

The FP of the hES cardiomyocyte networks was recorded with the self-made on-chip MEA system at a sampling rate of 50 kHz at 37 °C in 5% CO_2_ (Figure 3A). The FP signals on a microelectrode were amplified and digitally recorded for waveform analysis to evaluate the effect of E-4031 on the inward/outward cell membrane current changes.

The time course of the stepwise E-4031 administration procedure is illustrated in Figure 3B. The FPs of cardiomyocytes on each microelectrode were measured at seven different solution states in MEA chips; Free (only culture medium), Control (0.1% PBS dose), Low (1 nM E-4031 dose), Medium (10 nM E-4031 dose), High (100 nM E-4031 dose), Very high (1 μM E-4031 dose) and finally Wash-out (only culture medium). The concentration of E-4031 (dissolved in PBS) in the culture medium was increased stepwise by exchanging half culture medium in MEA chips. FP waveforms were recorded during the last 5 minutes of 10 min of drug exposure at each concentration, and the last 50 waveforms of the obtained data were adopted and analyzed.

Figure 3C exhibits an example of FP waveform changes in a large square sheet cardiomyocyte network during the stepwise E-4031 administration procedure. The first peak of the inward current of depolarization, which is mainly caused by the opening of sodium ion channels (red arrowheads in Figure 3C), and the last peak of the outward current of repolarization, which is mainly caused by the opening of potassium ion channels (green arrowheads in Figure 3C), were observed in the FP waveforms at Free, Control, Low, Medium and Wash-out states. The FP duration (FPD) prolongation, which is mainly caused by the inhibition of the opening of potassium ion channels such as hERG ion channels (an increase of the distance between the red and green arrowheads), was observed at Medium state (10 nM E-4031 dose). The fibrillation-like fluctuated waveforms were observed at a High (100 nM E-4031 dose) state. At the Very high (1 μM E-4031 dose) states, arrest of FP waveforms was observed (Figure 3C). These tendencies of the FP waveforms at each state from Control to Very high were observed similarly in large square sheet cardiomyocyte networks (n = 3).

Similarly to our previous reports [11,16,17], we also analyzed the stability of inter-spike interval (ISI) and FPD from each waveform in detail as indices to evaluate the toxic effect of the drug and calculated CV of ISI (Equation (2)) and STV of FPD (Equation (3)) as the evaluation of its fluctuation, respectively. Each value was treated as an expected value with the smallest value of STV of FPD on the electrode at the state of Control in each network, and the last 50 waveforms of the measured data were the target of evaluation. As shown in Figure 3D, a Poincaré plot, a kind of recurrence plot used to quantify self-similarity in FPDs and visualize their tendency of fluctuations in the graphs. To demonstrate the effectiveness of a Poincaré plot, we adopted the FPDs of the most fluctuated small sheet cardiomyocyte network data as an example. As shown in the blue trace of the FPD plotting, the fluctuation of FPDs in a small sheet at Control was very stable. In contrast, the plot of the FPDs at Medium state fluctuated significantly, indicating the neighboring FPD prolongation fluctuated. The STV of FPD is important to indicate these tendencies as values, 3 ms for Control and 11 ms for Medium state. We use STV of FPD to evaluate the potential difference of hERG ion channel response stabilities in three types of cardiomyocyte network geometry.

These large square sheet cardiomyocyte network results suggest that FPD prolongation was observed as a result of the appropriate effect of E-4031 as an hERG ion channel blocker in cardiomyocyte networks, as shown in the Medium state (10 nM E-4031 dose). Furthermore, when we add E-4031 to the High state (100 nM E-4031 dose), the majority of hERG ion channels were blocked by E-4031, resulting in weak and high-frequency depolarization and a fibrillation-like fast ISI waveform. Finally, at the very high state (1 μM E-4031 does), the inward/outward FP waveform disappeared because of the ion channel blocking, considered to be arrested.

In fact, the hERG current $IC_{50}$ of E-4031 was reported to be 7.7–14 nM [13,14], and the $EC_{50}$ of FPD was reported to be 5 nM [15]. Our results showed a similar tendency of E-4031 concentration dependence of hERG blocking to these previous studies. In our previous study, which examined the effect of E-4031 by patch-clamping the same cells as in this study, action potential duration of hES cells was prolonged with 10 nM concentration of E-4031 and was shortened with 100 nM concentration E-4031 [12]. These results also suggest that the tendency of FP waveform changing of a large square sheet of cardiomyocyte network by E-4031 was similar to the conventional in vitro patch-clamp measurement.

### 4.3. Effect of hERG Ion Channel Blocker E-4031 on Beating Inter-Spike Intervals of Three Type Cardiomyocyte Networks

**Figure 4:**
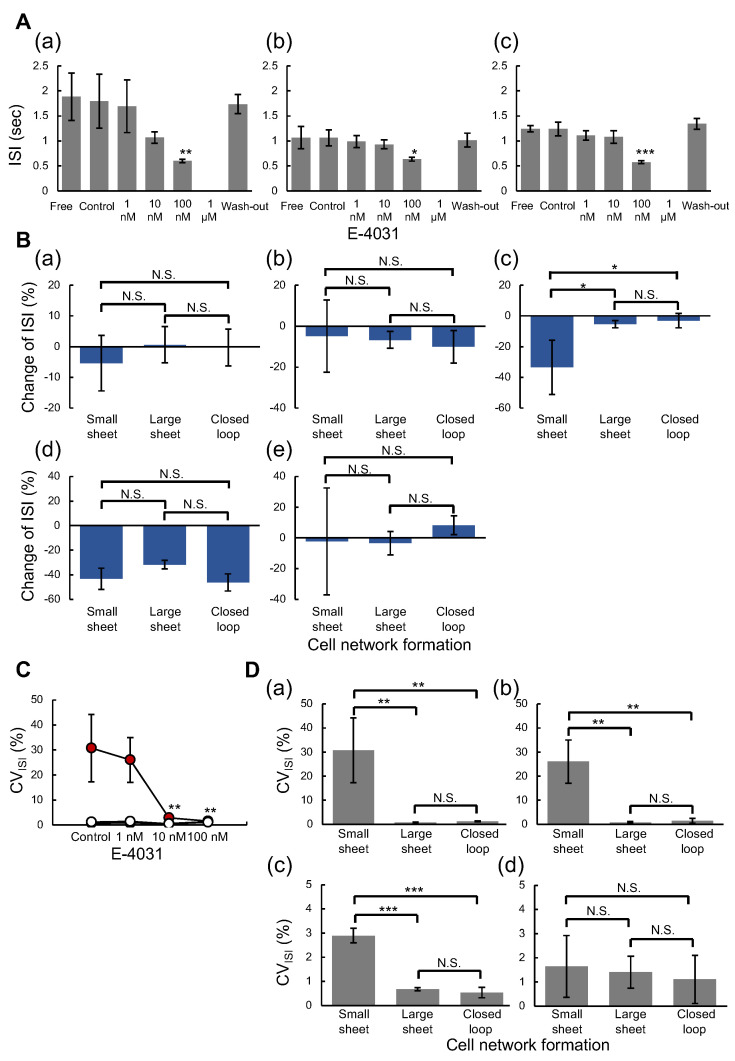
**Evaluation of hERG ion channel blocking on beating intervals in three type cardiomyocyte network patterns.** (**A**): E-4031 concentration dependence of inter-spike intervals (ISIs) in three type cardiomyocyte networks: (**a**) small sheet (n = 3), (**b**) large square sheet (n = 3) and (**c**) square closed-loop-shaped sheet (n = 3). Bars, mean values. Error bars, S.D. Dunnett’s multiple comparison test, * *p* < 0.05, ** *p* < 0.01, and *** *p* < 0.001 vs. Control state. (**B**): Change in ISI in three cardiomyocyte network patterns: (**a**) Free to Control (n = 3 each), (**b**) Control to Low (n = 3 each), (**c**) Low to Medium (n = 3 each), (**d**) Medium to High (n = 3 each) and (**e**) Free to Wash-out (n = 3 each). Bars, mean values. Error bars, S.D. Tukey’s multiple comparison test, * *p* < 0.05. (**C**): Coefficient of variability (CV) of ISI values against E-4031 concentration. The red-filled circles, black-filled circles, and open circles indicate the mean CV values of the small sheet, large square sheet, and square closed-loop-shaped sheet group, respectively (n = 3 each). Dunnett’s multiple comparison test, ** *p* < 0.01 vs. Control state. (**D**): CV of ISI in three cardiomyocyte network patterns: (**a**) Control state (n = 3 each), (**b**) Low state (n = 3 each), (**c**) Medium state (n = 3 each) and (**d**) High state (n = 3 each). Bars, mean values. Error bars, S.D. Tukey’s multiple comparison test, ** *p* < 0.01 and *** *p* < 0.001.

To evaluate whether the effect of E-4031 on the beating intervals varies with the network pattern of cardiomyocytes, we analyzed the inter-spike interval (ISI) of these networks in each concentration of E-4031. Figure 4A shows the ISI changes against E-4031 concentration in three cardiomyocyte network patterns: (a) small sheet (n = 3), (b) large square sheet (n = 3) and (c) square closed-loop-shaped sheet (n = 3). The mean ISI significantly shortened at 100 nM E-4031 dose to compare with the Control state in all groups. Moreover, all cardiomyocyte networks arrested the FP waveforms at 1 μM E-4031 dose. Figure 4B compares the mean values of ISI changes between groups. The ISI change (%), ISI at *i* state to ISI at *j* state, was calculated $\left(ISI_{j}-ISI_{i}\right)/ISI_{i}\times 100$ (%) where $ISI_{i,j}$ represents the mean ISI of the cardiomyocyte network at i,j state. The (a), (b), (c), (d), and (e) show the change of ISI from Free (only culture medium) state to Control (0.1%PBS dose) state, Control to Low (1 nM E-4031 dose), Low to Medium (10 nM E-4031 dose), Medium to High (100 nM E-4031 dose), and Free to Wash-out (only culture medium), respectively. There was no tendency for ISI changes and no significant differences between the groups in (a), (b), and (e). The change of ISIs from Low state to Medium state in small sheet, large square sheet, and closed-loop sheet were −33.5 ± 17.7%, −5.3 ± 2.7% and −3.1 ± 4.6%, respectively, and significantly greater for the small sheet group than for the large sheet group and the closed-loop group. The ISI change from Medium state to High state was negative (−54.1 to −27.9% range) in all three cardiomyocyte networks, and no significant difference in ISI changes were observed between groups.

To evaluate the effect of the E-4031 on the fluctuation of ISI, we calculated CV (%) of ISI (Equation (2)). Figure 4C shows the CV of ISI against E-4031 concentration. The mean CV of ISI was significantly decreased only in the small sheet group at 10 nM and 100 nM E-4031 to compare with the Control state (red-filled circles). Figure 4D compares the mean CV of ISI between groups: (a) Control state, (b) Low state, (c) Medium state, and (d) High state, respectively. The mean CV of ISI was significantly larger for the small sheet group than for the large square sheet group and the closed-loop group at Control, Low, and Medium states. The CV of ISI of the small sheet group, the large square sheet group, and the closed-loop group at the Control state were 30.7 ± 13.4%, 0.7 ± 0.2% and 1.3 ± 0.1%, respectively. At the Medium state, these values were 2.9 ± 0.3%, 0.7 ± 0.1%, and 0.5 ± 0.2%, respectively. However, there were no significant differences in CV of ISI between groups at the High state.

**Figure 5:**
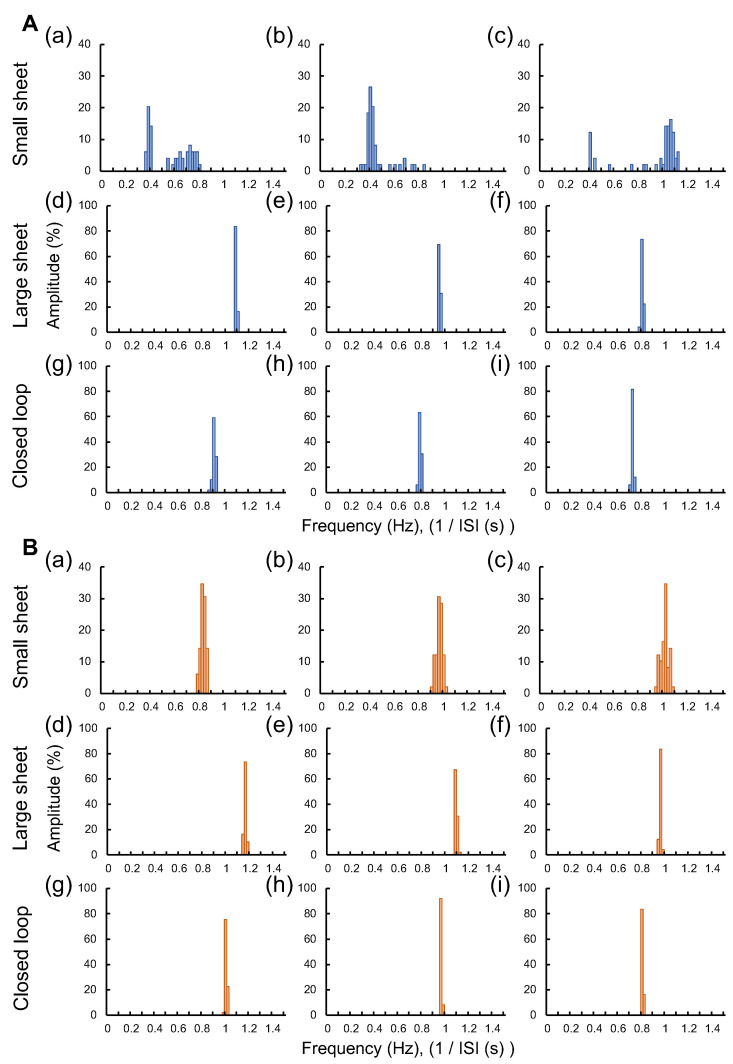
**The beating frequency distribution of three type cardiomyocyte network samples at Control and Medium states.** (**A**): The distribution of beating frequency (1/ISI, Hz) of three samples in Control state: (**a**–**c**) small sheet group, (**d**–**f**) large sheet group, and (**g**–**i**) closed-loop group. (**B**): The distribution of beating frequency in Medium state: (**a**–**i**) were the same cardiomyocyte network samples as shown in **A**(**a**–**i**).

To identify the cause of this ISI tendency, we plotted the beating frequency distribution of individual samples of three types of cardiomyocyte networks in Figure 5A,B show the distribution of beating frequency (Hz) of three type cardiomyocyte networks at Control state and Medium state, respectively. Three samples of small sheet group (a–c), large sheet group (d–f), and closed-loop group (g–i) were described.

In the Control state, the two different peaks of beating frequency were observed in small sheet group samples (Figure 5A(a–c)). Two peaks of ISIs were at 0.38 and 0.72 Hz, 0.40 and 0.68 Hz and 0.40 and 1.06 Hz in (a), (b), and (c), respectively. In contrast, single sharp peaks of beating frequency were observed in the large sheet group and closed-loop group and were at 0.72–1.08 Hz in (d) to (i).

At the Medium state, the single peak of beating frequency was observed in all cardiomyocyte network samples (Figure 5B(a–i)). However, the distributions of beating frequency in small sheet group samples were still wider than the other two types.

These results suggest that E-4031 is effective as an antiarrhythmic agent in small sheets, which were inherently unstable in beating, and that some degree of regular beating was observed under 10 nM E-4031 administration. It is consistent with the previous report that the spontaneous beating variability disappeared and became aligned to around 1 Hz at the E-4031 dose in cell populations with different frequencies [18]. In our previous study, the distribution of ISI became narrower, and the beating tended to be more stable and organized when cells were clustered than when single cells were used in the same hES cells [19].

Therefore, the shape of the small sheet has the advantage of preparing the fluctuation model to examine the antiarrhythmic ability of compounds. In contrast, large cardiomyocyte networks both in 2D sheets and closed-loop sheets maintained coordinated beating intervals even without E-4031, regardless of their shape differences, indicating that the large cell number model is not suitable for antiarrhythmic screenings.

### 4.4. Effect of hERG Ion Channel Blocker E-4031 on Field Potential Duration Time of Cardiomyocyte Networks

**Figure 6:**
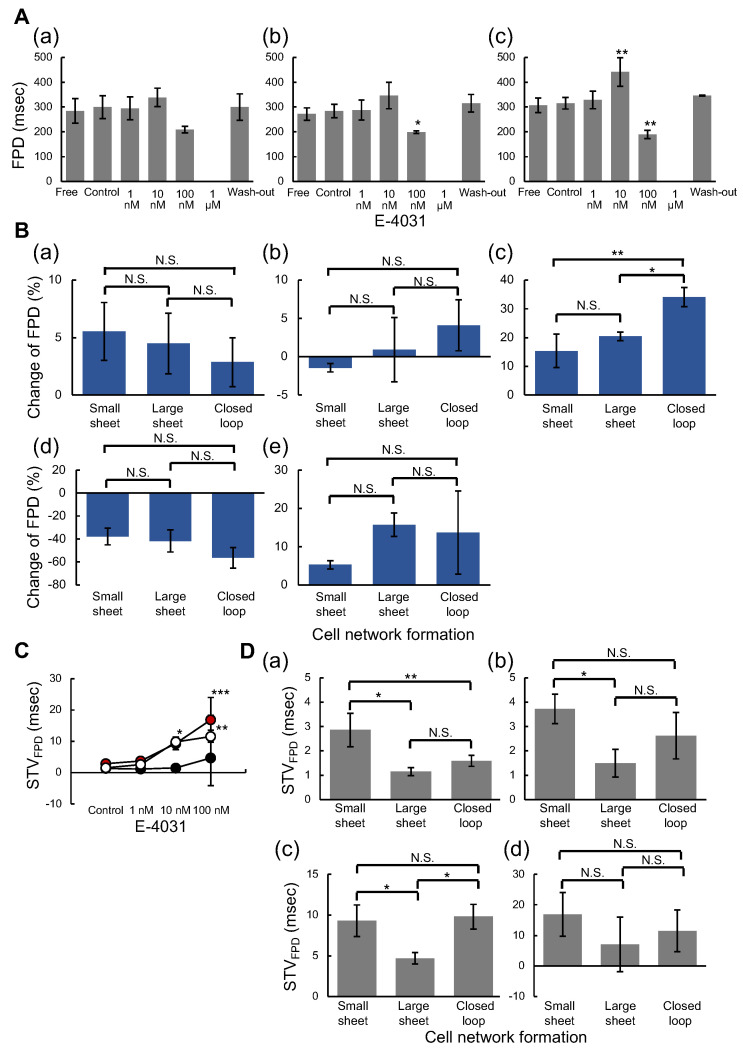
**Evaluation of hERG ion channel blocking on field potential duration time prolongation in three types of cardiomyocyte network patterns.** (**A**): E-4031 concentration dependence of field potential duration (FPD) time: (**a**) small sheet (n = 3), (**b**) large square sheet (n = 3), and (**c**) large square closed-loop sheet (n = 3). Bars, mean values. Error bars, S.D. Dunnett’s multiple comparison test, * *p* < 0.05 and ** *p* < 0.01 vs. Control state. (**B**): Change in FPD in three cardiomyocyte network patterns: (**a**) Free to Control (n = 3 each), (**b**) Control to Low (n = 3 each), (**c**) Low to Medium (n = 3 each), (**d**) Medium to High (n = 3 each), and (**e**) Free to Wash-out (n = 3 each). Tukey’s multiple comparison test, * *p* < 0.05 and ** *p* < 0.01. (**C**): Short-term variability (STV) of FPD values against E-4031 concentration. The red, black, and open circles indicate mean STV values of the small, large square, and square closed-loop groups, respectively (n = 3 each). Dunnett’s multiple comparison test, * *p* < 0.05, ** *p* < 0.01, and *** *p* < 0.001 vs. Control state. (**D**): STV of FPD in three cardiomyocyte network patterns: (**a**) Control state (n = 3 each), (**b**) Low state (n = 3 each), (**c**) Medium state (n = 3 each), and (**d**) High state (n = 3 each). Bars, mean values. Error bars, S.D. Tukey’s multiple comparison test, * *p* < 0.05 and ** *p* < 0.01.

We examined the effect of E-4031 on the field potential duration (FPD) in three types of cardiomyocyte networks against E-4031 concentration increase. Figure 6A shows the FPD change against E-4031 concentration in three cardiomyocyte network patterns: (a) small sheet (n = 3), (b) large square sheet (n = 3), and (c) square closed-loop sheet (n = 3), respectively. The mean FPD were increased at 10 nM E-4031 dose, decreased at 100 nM E-4031 dose and finally arrested at 1 μM E-4031 dose. These tendencies were observed in all three types of groups. However, the mean FPD was significantly prolonged at 10 nM E-4031 dose than the Control state only in the closed-loop group. The mean FPD was significantly shortened at 100 nM E-4031 dose than the Control state in the large sheet and the closed loop groups.

Figure 6B compares FPD changes between groups. The FPD change (%) was also calculated in the same way as ISI change: (a) Free (only culture medium) state to the Control (0.1%PBS dose) state, (b) Control state to Low (1 nM E-4031 dose) state, (c) Low state to Medium (10 nM E-4031 dose) state, (d) Medium state to High (100 nM E-4031 dose) state, and (e) Free state to Wash-out (only culture medium) state, respectively. The mean FPD changes from Free state to Control state in the small sheet group, the large square sheet group, and the square closed-loop sheet group were 5.5 ± 2.5%, 4.5 ± 2.6%, and 2.9 ± 2.1%, respectively. The mean FPD change from Low to Medium state was significantly greater for the closed-loop group than for the small and large sheet groups. Their values in the small sheet group, the large square sheet group, and the closed-loop group were 15.4 ± 5.8%, 20.4 ± 1.5%, and 34.1 ± 3.3%, respectively. However, the FPD from Medium to High state changed negatively (−62.5 to −29.6% range) in all cardiomyocyte networks.

To evaluate the effect of E-4031 on FPD fluctuation, we applied STV (ms) of FPD for comparison of three types of cardiomyocyte networks (Equation (3)). Figure 6C shows the relationship between the mean STV of FPD and the concentration of E-4031. The red-filled, black-filled, and open circles indicate the mean STV values in small, large square sheet and closed-loop groups. The mean STV of FPD was increased as the E-4031 concentration rose in each group; they significantly increased only in the closed-loop group at 10 nM E-4031 dose compared to the Control state. The STV of FPD in the small sheet and closed-loop groups at 100 nM E-4031 were significantly increased compared to the Control state. Figure 6D shows the comparison of mean STV of FPD: (a) Control state, (b) Low state, (c) Medium state, and (d) High state. The mean STV of FPD was significantly larger for the small sheet group than for the large sheet group and the closed loop group at the Control state. The STV of FPD in the small sheet, large sheet, and closed-loop groups were 2.9 ± 0.7 ms, 1.2 ± 0.2 ms, and 1.6 ± 0.2 ms, respectively. At the Medium state, the STV of FPD was significantly smaller for the large sheet group than for the small and closed-loop groups. The values in the small sheet group, the large sheet group, and the closed-loop group were 9.3 ± 1.9 ms, 4.7 ± 0.7 ms, and 9.8 ± 1.5 ms, respectively.

These results indicate that the closed-loop cardiomyocyte network was the most sensitive to the hERG channel-blocking effect of E-4031. Comparing the large and the closed-loop sheets, only the closed-loop sheet was significantly prolonged at 10 nM E-4031, suggesting that these differences were not due to the cell number but the geometry of cells. Furthermore, significant differences in both FPD and STV of FPD were observed between groups at 10 nM E-4031 dose for only the closed-loop sheet when compared to the Control state, suggesting that the sensitiveness of in vitro ion-channel screening of hERG response to E-4031 depends on the geometry of cardiomyocyte networks.

**Figure 7:**
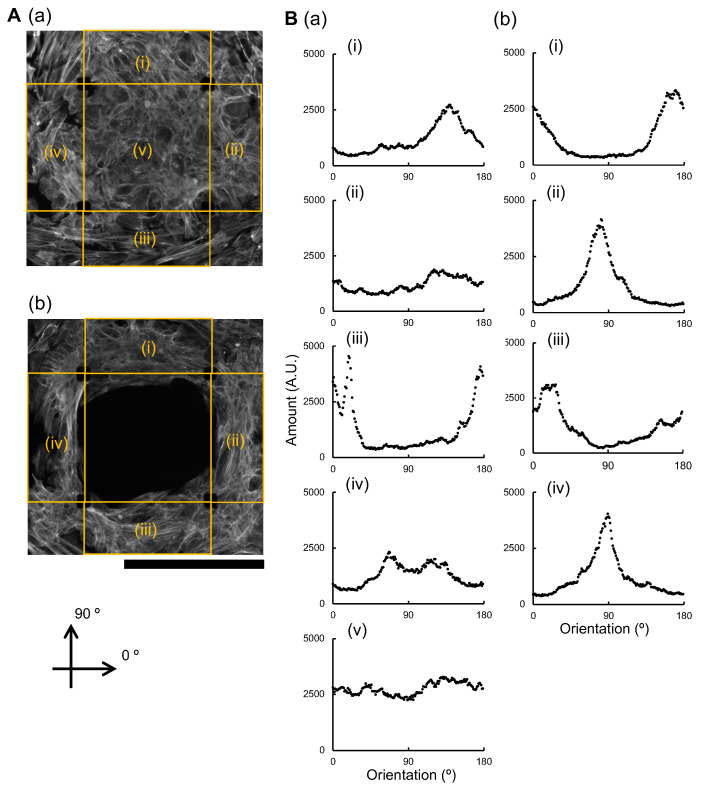
**Cell orientation analysis of cardiomyocytes in the large square sheet and closed-loop sheet microchambers.** (**A**): Fluorescent micrographs of hES cardiomyocytes in the large square agarose microchamber (**a**) and closed-loop agarose microchamber (**b**) on an MEA chip seven days after cultivation started. Rhodamine Phalloidin stained actin filaments in cardiomyocytes. Bar, 0.5 mm. (**B**): Cell orientation distribution in large square sheet (**a**) and square closed-loop-shaped sheet (**b**). Acquired images were divided into five (**a**) and four (**b**) areas, and their cell orientation distribution estimated by actin filament orientations in each cell were analyzed. (**i**–**iv**) represent the four sides of the large square sheet, and closed-loop-shaped sheet, (**v**) is the inner central area of the large square sheet.

We analyzed the orientation distribution of the component cardiomyocytes in each sheet to examine the origin of the difference in FPD prolongation behaviors in the large square sheet and the square closed-loop-shaped sheet. Figure 7A shows the fluorescent micrographs of stained actin filaments of the large square sheet cardiomyocyte network (a) and the square closed-loop-shaped sheet cardiomyocyte network (b). As shown in those images, the actin filament distributions can estimate the orientations of component cardiomyocyte cells. Figure 7B shows the distribution of orientation of component cells in the large square sheet cardiomyocyte network (a) and the large closed-loop-shaped sheet cardiomyocyte network (b). As shown in the graph, the orientation of the component cells in the large square sheet cardiomyocyte network did not align in (ii), (iv), and (v) in five regions. Significantly, there is no orientation observed at the inner central region. In contrast, those in the large closed-loop-shaped sheet cardiomyocyte network tended to align in all (i)–(iv) areas.

These results suggest that the proper confinement structures gain the alignment of component cell orientations, resulting in coordinated enhanced synchronous behaviors in those cardiomyocyte networks. As described in the previous reports [20,21,22], stretch-induced Calcium release mechanism in muscle contraction control can be coupled to the membrane potentials. Hence, the alignment of component cardiomyocytes in the same direction of contraction may lead to a more stable response of single cells in the population, and also, the fluctuation of neighboring cells might be suppressed.

In recent years, culture methods have been developed to reproduce the function of the heart in vivo on a chip that controls the orientation of cardiomyocytes. It has been reported that there were differences in the expression of proteins such as Cx43 between cells that were cultured with and without aligned cell orientation [23], and that the contractility was higher in sheets of myocardium that were cultured with aligned cell orientation than in those that were not [24]. We have not elucidated the exact cause or mechanism of the hypersensitivity of the closed-loop-shaped cardiomyocyte networks to the drug concerning FPD prolongation in this paper yet. However, one possibility is that the closed-loop-shaped cardiomyocyte network is more aligned than the large square sheet in terms of the orientation of its constituent cells, which may be related to its drug responsiveness.

### 4.5. Importance of Geometry-Controlled Cardiomyocyte Network Analysis Using at Least Three Geometry of Network Patterns

Previous studies on the drug response in the spatial arrangement of cardiomyocytes reported that TdP waveforms were successfully observed in a population in which heterogeneous cells were mixed and cultured in two dimensions [16,25,26] and in three dimensions [27]. However, there has been no previous report about the geometrical dependence of cardiomyocyte ion channel drug response on the size and shape of the cardiomyocyte networks in confined structures.

The results of this paper indicate the importance of strict control of cell number and cell network geometry for reproducible and stable results even for evaluating in vitro hERG ion channel responses. Our results suggest the usefulness of comparing three types of cardiomyocyte networks, small sheet, large square sheet, and closed-loop-shaped sheet, simultaneously on a chip to prevent overestimation and underestimation of in vitro risk assessment in cardiotoxicity. As summarized in Table 1, these three shapes of cardiomyocyte networks have complementary characteristics among them; small sheets can reveal the instability of cell-to-cell relationships, especially in the beating intervals; large sheets can form the most stable and reproducible responses of idealized cardiomyocytes; and closed-loop sheets are most sensitive to the hERG blocking compound E-4031 as the significant increase in STV of FPDs, or the prolongation and fluctuation of FPDs. The small sheet cardiomyocyte networks are suitable for evaluating the drug’s antiarrhythmic function because the small number of cardiomyocyte cells have more fluctuated in their beatings and can be used to evaluate the effectiveness of antiarrhythmic compounds. For evaluating QT prolongation, the closed-loop cardiomyocyte networks should be used for predictive toxicity from the viewpoint of the safer side because they are the most sensitive to hERG-blocking compounds, even when the other two geometries did not indicate FPD prolongation. The use of the large sheet form cardiomyocyte networks as a control group of idealized cardiomyocytes to reduce the risk of over-assessing the side effects of drugs.

In our previous report, we demonstrated drug response in the lined-up cardiomyocyte networks [17]. In the report, we found the drug responsiveness of conduction time in the two-dimensional lined-up cardiomyocyte networks and the importance of the strict width control of cardiomyocyte networks for stable propagation of excitation conduction. This time, we focused on the hERG ion channel response in three geometry of cardiomyocyte networks. The results reported in this paper indicate the importance of cell network size and shape control for acquiring reliable results even for the in vitro ion channel evaluations. To unveil the relevance between these in vitro results to in vivo pathophysiological processes, we need to add more detailed experimental results such as more detailed geometry dependence, cell type dependence, and also the effects in other types of compounds; astemizole and terfenadine; multi-channel blockers, quinidine, lidocaine, and flecainide; sodium channel blocker, and verapamil; calcium channel blocker. Although we cannot address the detailed mechanism of the phenomenon in this paper yet, we can present the fact that different spatial arrangements of cells exhibit different drug responses. These results suggest that the designing of simultaneous evaluation of varying cardiomyocyte networks of multiple shapes allows a more accurate assessment of the cardiotoxicity of drugs.

## 5. Conclusions

The spatial arrangement dependence of cardiomyocyte network response against hERG ion channel blocker E-4031 has been examined. Three types of cardiomyocyte networks, small sheet, large square sheet, and square closed-loop-shaped sheet, were formed in the agarose microchambers fabricated on a multi-electrode array chip. We found different responses to the administration of drugs among the three groups. In small sheets, the fluctuated beating of cardiomyocytes in the networks was stabilized with 10 nM E-4031. In the closed-loop cardiomyocyte networks, the FPD was prolonged with 10 nM E-4031, even though the other two sheets remained normal at this concentration. In the large sheet group, the cardiomyocyte networks were most durable and robust against E-4031 among the three geometries of cardiomyocyte networks. These results indicate that the difference in cardiomyocyte network patterns influences the in vitro ion channel drug evaluation to overestimate or underestimate the risks depending on the geometry of their network patterns, suggesting the importance of controlling the cardiomyocyte network geometry for reproducible and reliable results, or comparing at least three types of cardiomyocyte networks simultaneously as described in this report, to allow for more accurate assessment of drug responses.

## Figures and Tables

**Table 1:** Summary of the effect of E-4031 on three geometry of cardiomyocytes.

	Small Sheet	Large Square Sheet	Square Closed-Loop-Shaped Sheet
ISI	+	−	−
FPD	−	−	+
STV of FPD	−	−	+

−: Stable, +: Sensitive.

## Data Availability

Not applicable.
